# Clinical and microbiological characteristics of *Cryptococcus gattii* isolated from 7 hospitals in China

**DOI:** 10.1186/s12866-020-01752-4

**Published:** 2020-03-30

**Authors:** Liang Jin, Jing-Rong Cao, Xin-Ying Xue, Hua Wu, Li-Feng Wang, Ling Guo, Ding-Xia Shen

**Affiliations:** 1 Medical laboratory center, First Medical Center of Chinese PLA General Hospital & Medical School of Chinese PLA, No.28 Fuxing Road, Haidian District, Beijing, 100853 China; 2 Department of Clinical Laboratory, the First Hospital of Qinhuangdao, Qinhuangdao, 066000 China; 3 Department of Clinical Laboratory, Xuanwu Hospital of Capital Medical University, Beijing, 100053 China; 4 Department of Respiratory and Critical Care Medicine, the Affiliated Beijing Shijitan Hospital of Capital Medical University, Beijing, 100038 China; 5 Department of Clinical Laboratory, Hainan General Hospital, Haikou, 570311 China

**Keywords:** *Cryptococcus gattii*, Genotype, Antifungal agents, Spectrum, Differential protein

## Abstract

**Background:**

Infection, even outbreak, caused by *Cryptococcus gattii* (*C. gattii*) has been reported in Canada and the United States, but there were sparsely-reported cases of *C. gattii* in China. Our interest in occurrence, clinical manifestation, laboratory identification and molecular characterization of Chinese *C. gattii* strains leads us to this research.

**Results:**

Out of 254 clinical isolates, initially identified as *Cryptococcus neoformans* (*C. neoformans*), eight strains were re-identified as *C. gattii*. Multi-locus sequence typing (MLST) showed genotype VGI accounted for the most (6 / 8), the other two strains were genotype VGII (VGIIa and VGIIb respectively) with 3 specific spectra of molecular weight about 4342, 8686, 9611 Da by MALDI-TOF MS. The minimal inhibitory concentrations (MICs) of Fluconazole with Yeast one was 2~4 times higher than that with ATB fungus 3 and MICs of antifungal agents against VGII strains were higher than against VGI strains. Comparative proteome analysis showed that 329 and 180 proteins were highly expressed by *C. gattii* VGI and VGII respectively. The enrichment of differentially expressed proteins was directed to Golgi complex.

**Conclusions:**

Infection by *C. gattii* in China occurred sparsely. Genotype VGI was predominant but VGII was more resistant to antifungal agents. There was significant difference in protein expression profile between isolates of VGI and VGII *C. gattii*.

## Background

*Cryptococcus gattii* (*C. gattii*), one of the most common pathogenic cryptococcal species, primarily infects immunocompetent hosts, in contrast to *Cryptococcus neoformans* (*C. neoformans*), which mainly infects immunocompromised individuals [1, 2]. Previous studies revealed that *C. gattii* infection was restricted geographically to tropical and subtropical regions [1–3]. However, outbreaks have been recorded in temperate regions, such as British Columbian of Canada and Pacific Northwest of the United States [4, 5]. The majority of Chinese people reside in temperate and subtropical regions with climates suitable for fungal growth and the incidence of cryptococcal infection increased in recent years, unfortunately, little investigation has been carried out on this organism in China [6]. The objectives of the present study were to determine the frequency of *C. gattii* infection, to investigate clinical and microbiological characterization, and to analyze molecular features of the Chinese isolates.

## Results

### Identification and antifungal susceptibility profile of *C. gattii* isolates

**Fig. 1:**
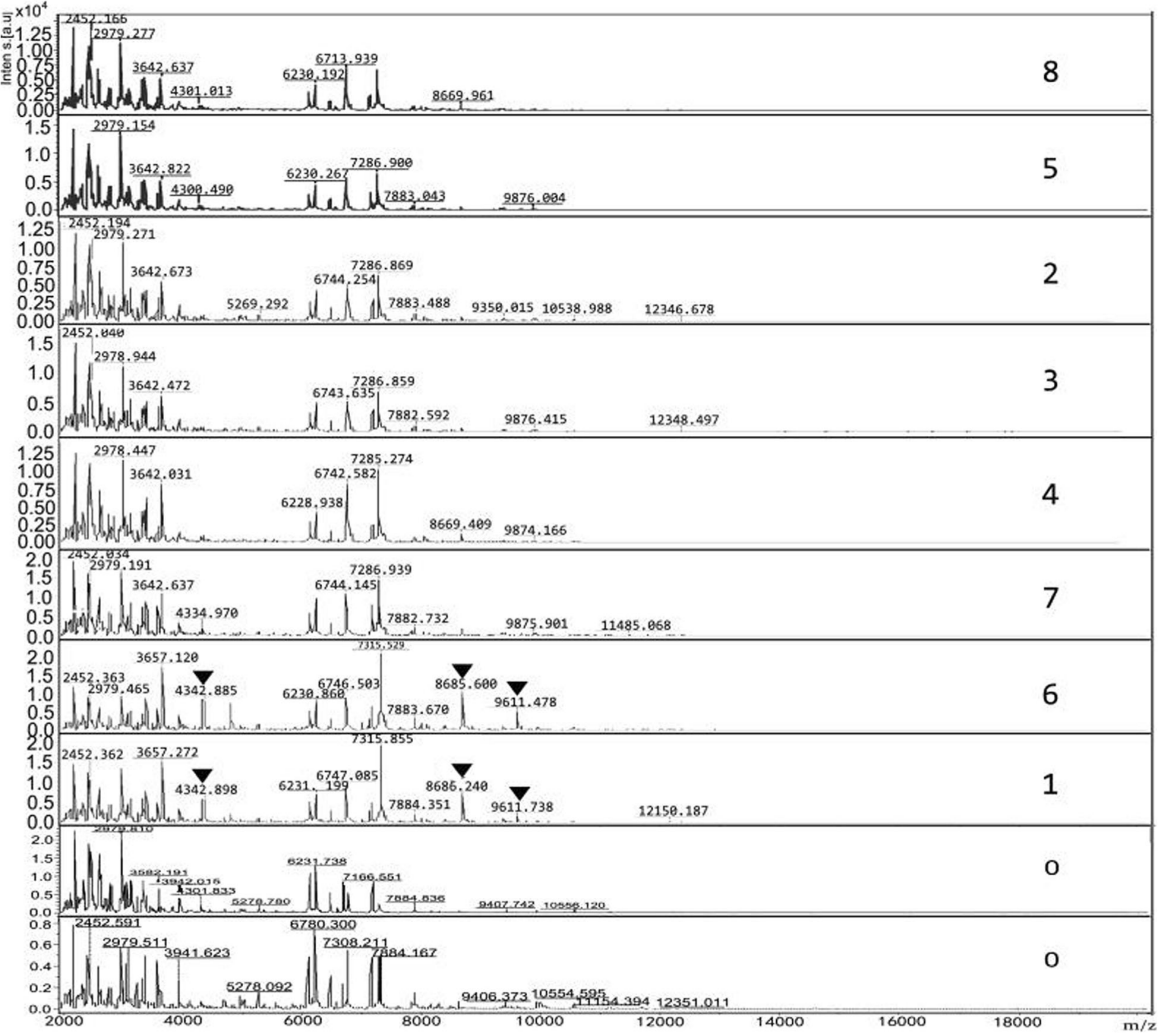
Mass spectra of 8 strains of *C. gattii* and 2 strains of *C.neoformans* by MALDI-TOF MS. Notes: Three specific spectra of *C. gattii* VGII with molecular weight of about 4342, 8686, 9611 Da were indicated as arrows; 0 represented *C.neoformans.*

**Fig. 2:**
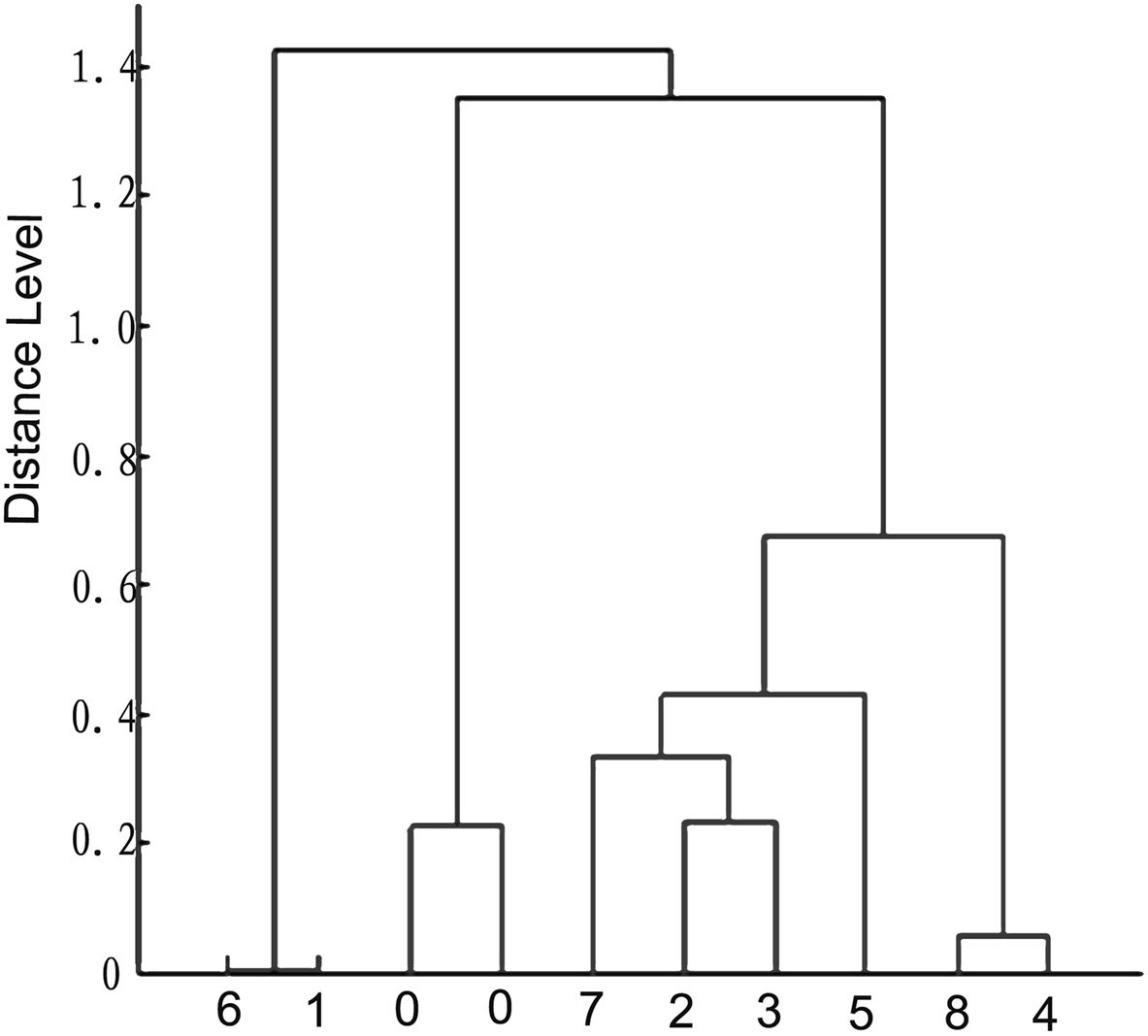
The dendrogram of 8 strains of *C. gattii* and 2 strains of *C.neoformans*

Among 254 collected Cryptococcal strains, eight showed blue pigmentation on canavanine glycine bromothymol blue (CGB) agar and were characterized as *C.gattii* by matrix-assisted laser desorption/ionization time-of-flight mass spectrometry (MALDI-TOF MS) with scores from 2.043 to 2.275, otherwise they were all identified as *C.neoformans* by VITEK 2 compact as seen in Table 1. The mass spectra obtained from eight *C. gattii* isolates were characterized by diverse spectra in the range between 2000 and 10,000 Da. Three specific spectra with molecular weight of about 4342, 8686, 9611 Da could be seen in two isolates of *C. gattii* VGII, they were absent in six isolates of *C. gattii* VGI, as shown in Fig.1. The dendrogram created by MALDI Biotyper divided eight isolates into two groups of *C. gattii* VGI and VGII as shown in Fig.2. The MICs of six antifungal drugs to eight strains of *C. gattii* were listed in Table 1.

**Table 1:** The geographical region of the isolation, species identification, antifungal susceptibility profile, genotype and sequence type of 8 *C. gattii* strains

Isolate No.	ID by VITEK 2 Compact	pigmentation on CGB agar	ID by MALDI-TOF MS	geographical region of the isolation	STs	Genotype	5-FU		AMB		FCA		ITR		VRC		PSZ
	(Identification rate)		(score)				a	b	a	b	a	b	a	b	a	b	b
1	*C. neoformans* (93%)	blue	C. gattii (2.190)	temperate regions	20	VGII	< 4	2	< 0.5	1	8	32	0.25	0.25	0.25	0.25	0.25
2	C. neoformans (90%)	blue	C. gattii (2.043)	tropical areas	106	VGI	< 4	0.5	< 0.5	0.25	1	4	< 0.125	0.06	0.06	0.06	0.06
3	C. neoformans (96%)	blue	C. gattii(2.275)	subtropical area	57	VGI	< 4	0.5	< 0.5	0.25	1	4	< 0.125	0.12	0.06	0.12	0.12
4	C. neoformans (99%)	blue	C. gattii (2.092)	subtropical area	197	VGI	< 4	1	< 0.5	0.25	2	4	< 0.125	0.06	< 0.125	0.06	0.12
5	C. neoformans (86%)	blue	C. gattii (2.118)	temperate regions	57	VGI	< 4	0.5	< 0.5	0.25	2	4	< 0.125	0.12	0.06	0.06	0.12
6	C. neoformans (91%)	blue	C. gattii (2.126)	tropical areas	7	VGII	< 4	2	< 0.5	0.5	8	128	0.25	0.25	0.25	0.25	0.5
7	C. neoformans (98%)	blue	C. gattii (2.188)	subtropical area	57	VGI	< 4	0.5	< 0.5	0.25	1	4	< 0.125	0.06	0.06	0.06	0.12
8	C. neoformans (99%)	blue	C. gattii (2.194)	temperate regions	161	VGI	< 4	1	< 0.5	0.25	2	4	< 0.125	0.12	0.06	0.06	0.12

Notes: a and b represented the method of ATB fungus 3 and Yeast one respectively; the abbreviations for antifungal drugs were: *5-FU* 5-Flucytosine, *AMB* Amphotericin B, *FCA* Fluconazole, *ITR* Itraconazole, *VRC* Voriconazole, *PSZ* Posaconazole

### MLST analysis and genotyping

**Fig. 3:**
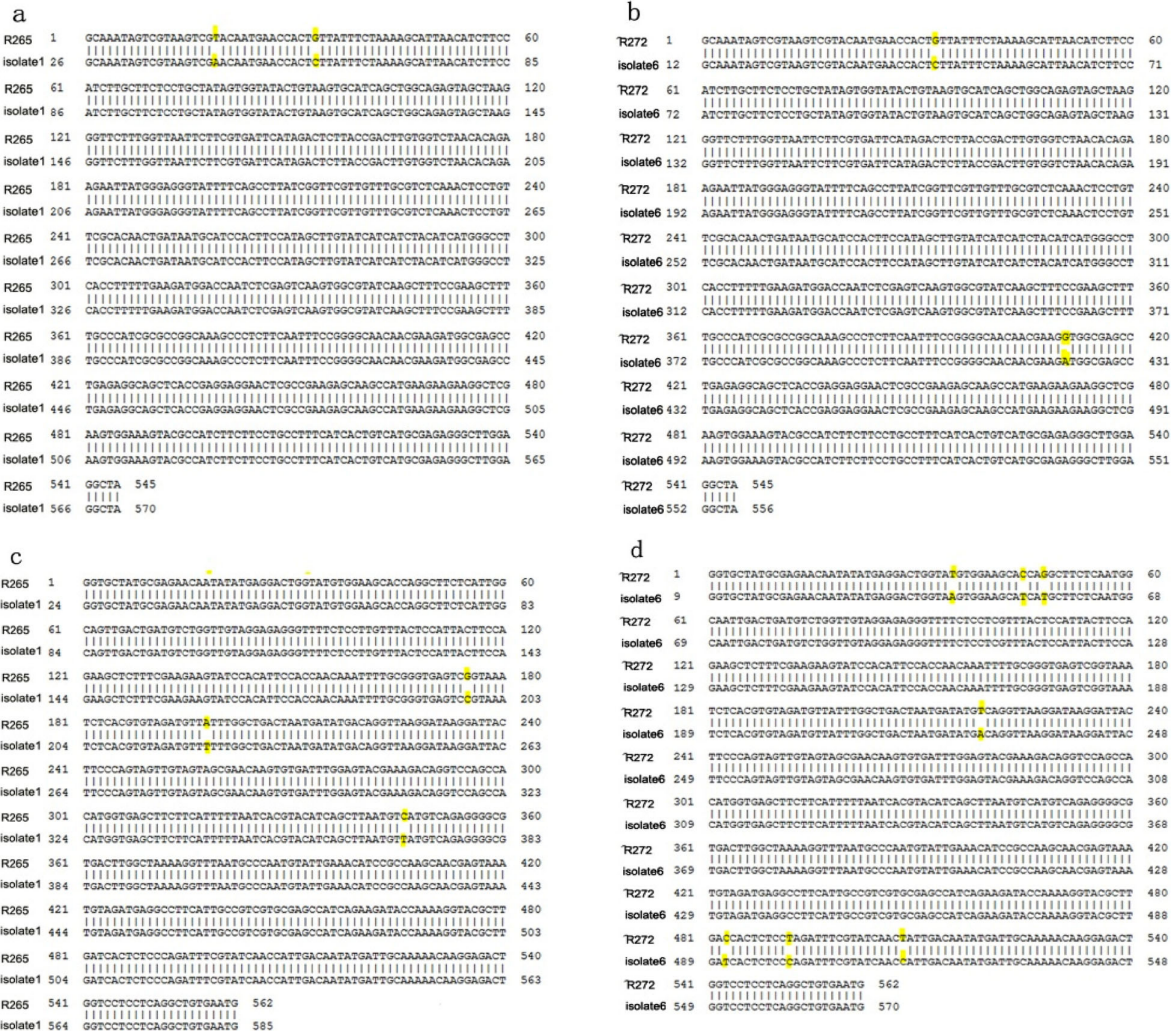
Gene mutations of 2 VGII strains (isolate number 1 and 6) in unlinked gene loci FTR1 and RAS1 compared with reference strains R265 and R272

MLST confirmed six strains of *C. gattii* VGI and two strains of *C. gattii* VGII as indicated in supplementary Table 1. The 2 strains of isolate number 1 and 6 with *C.gattii* genotype VGII could be subtyped into VGIIa and VGIIb respectively by further comparison with Vancouver Island reference stains of R265 and R272, as in supplementary Table 2. Some gene mutation existed in unlinked gene loci such as FTR1 and RAS1 as indicated in Fig.3.

### Clinical information of patients infected with *C. gattii*

As shown in Table 1, the eight strains of *C. gattii* were isolated from eight patients. Two patients with isolate numbers of 2 and 6 were from Hainan and Yunnan Province, which were recognized as tropical areas. Three patients with isolate number 3 (from Chongqing city), 4 and 7 (from Fujian Province) were recognized as being from subtropical area. The other three patients with isolate numbers of 1, 5 and 8 were from Shandong Province, Inner Mongolia Autonomous Region and Henan Province, which were recognized as temperate regions. Information of patient infected with isolate number 3 was unavailable, the other 7 patients were immunocompetent young male adults, with age range of 21~60 years old. Their clinical and laboratory information were summarized in Table 2.

**Table 2:** Clinical and laboratory information for patients infected with *C. gattii*

	1	2	4	5	6	7	8
Location (Province)	Shangdong	Hainan	Fujian	Neimeng	Yunnan	Fujian	Henan
isolation period of *C. gattii*	2014.11	2015.12	2014.04	2016.06	2016.07	2015.09	2016.08
Gender	Male	Male	Male	Male	Male	Male	Male
Age-ranges (years old)	20–40	20–40	20–40	41–60	41–60	20–40	20–40
History (medical/contact)	No	No	No	No	No	No	No
Immunity	Normal	Normal	Normal	Normal	Normal	Normal	Normal
Fever	+	+	+	–	+	+	+
Headache	+	+	+	+	+	+	–
Nausea and Vomiting	–	+	+	+	–	–	–
Seizure	–	–	+	–	–	–	–
Neck stiffness	+	–	+	+	–	–	–
Kernig’s sign	+	+	+	–	–	–	–
Papilloedema	–	–	+	+	–	–	–
Lung CT	Irregular nodule with spicules and lobulation	Left lower lung mass	Normal	Left lung mass	Left lower lung consolidation	Multiple small nodules	Right upper lung mass
Brain MRI	Meningeal lesions	Normal	Meningeal enhancement	Left lacunar infarction	NA	Normal	Normal
Organism also found by lung biopsy	Yes	NA	NA	NA	NA	Yes	Yes
Blood culture	No growth	No growth	No growth	NA	NA	NA	No growth
Cryptococcal antigen titre in serum	1:1024	NA	NA	NA	NA	NA	1:640
CSF test:							
Pressure (mmH2O)	260	330	330	330	NA	200	normal
Glucose (mmol/L)	0.5	4.11	2.49	2.3	NA	2.22	normal
Protein (g/L)	1.57	0.71	0.35	0.73	NA	29	normal
Chloride (mmol/L)	111	144.7	117	102	NA	126.6	normal
White blood cells(×10^6^cells/L)	377	80	720	91	NA	368	normal
Ink staining	positive	positive	positive	positive	NA	NA	positive
Cryptococcal antigen titre	1:1024	NA	NA	NA	NA	NA	1:640
Organism cultured from	CSF	CSF	CSF	CSF/	sputum	CSF	sputum
Antifungal therapy regime	Flu+AmB	Flu+AmB	Flu+AmB	Flu+AmB	Flu+AmB	Flu+AmB	Flu+AmB
Improvement (follow-up)	alive	alive	alive	alive	alive	alive	alive
Neurological sequelae	No	No	Yes	No	No	No	No

Notes: + and – represented symptoms appeared and not appeared; NA represented data not available

### Differential protein analysis between *C. gattii* VGI and VGII

**Fig. 4:**
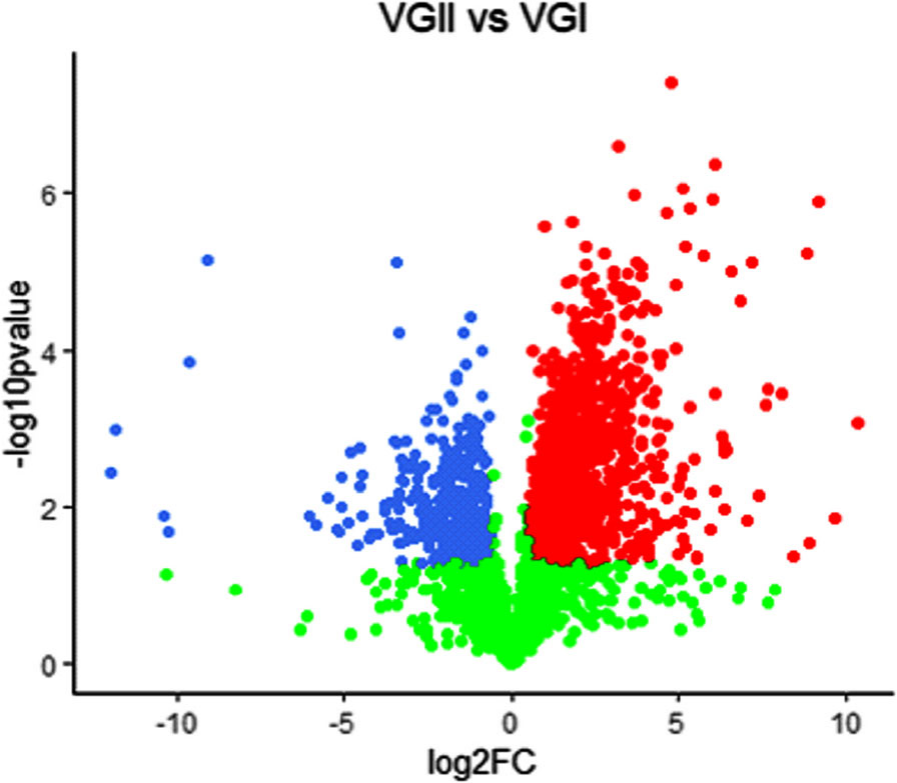
Volcanic map of identified proteins. Notes: The red and blue dot represented the profile for 329 and 180 highly expressed proteins of *C.gattii* VGI and VGII (p < 0.05); the green dot represented the proteins without significant difference

**Fig. 5:**
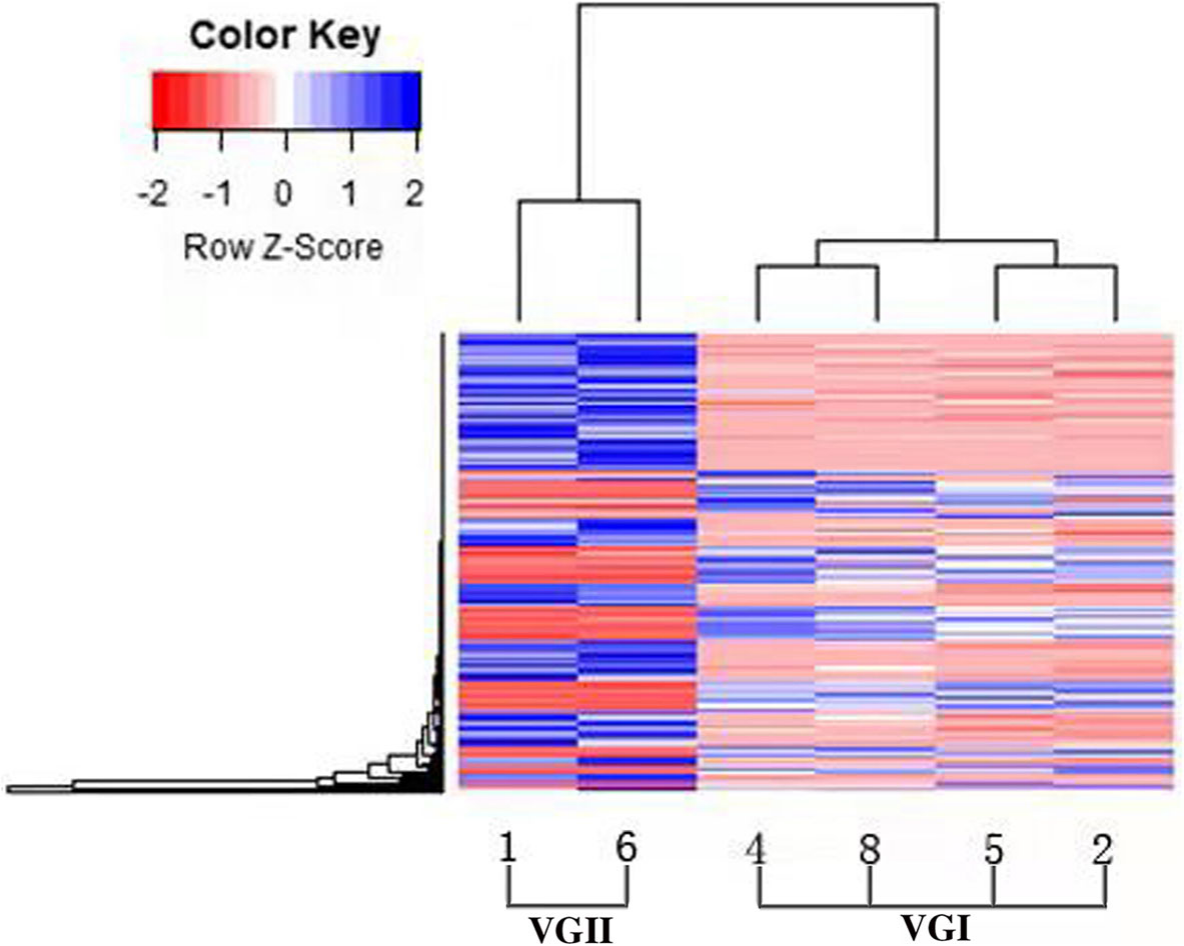
Clustering of differential expression proteins. Notes: Blue and red represented high and low expression proteins respectively

In this study, the proteome expression profiles of two strains of VGII and four strains of VGI *C. gattii* were created. A total of 3628 proteins were identified, of which, 2436 proteins were found in both VGII and VGI *C. gattii*, however, *C. gattii* genotype VGI and VGII possessed 774 and 418 specific proteins respectively. Comparative proteome analysis showed that 329 and 180 proteins were highly expressed in *C. gattii* VGI and VGII as shown in Fig.4. A cluster analysis of the differentially expressed proteins was shown in Fig. 5.

**Fig. 6:**
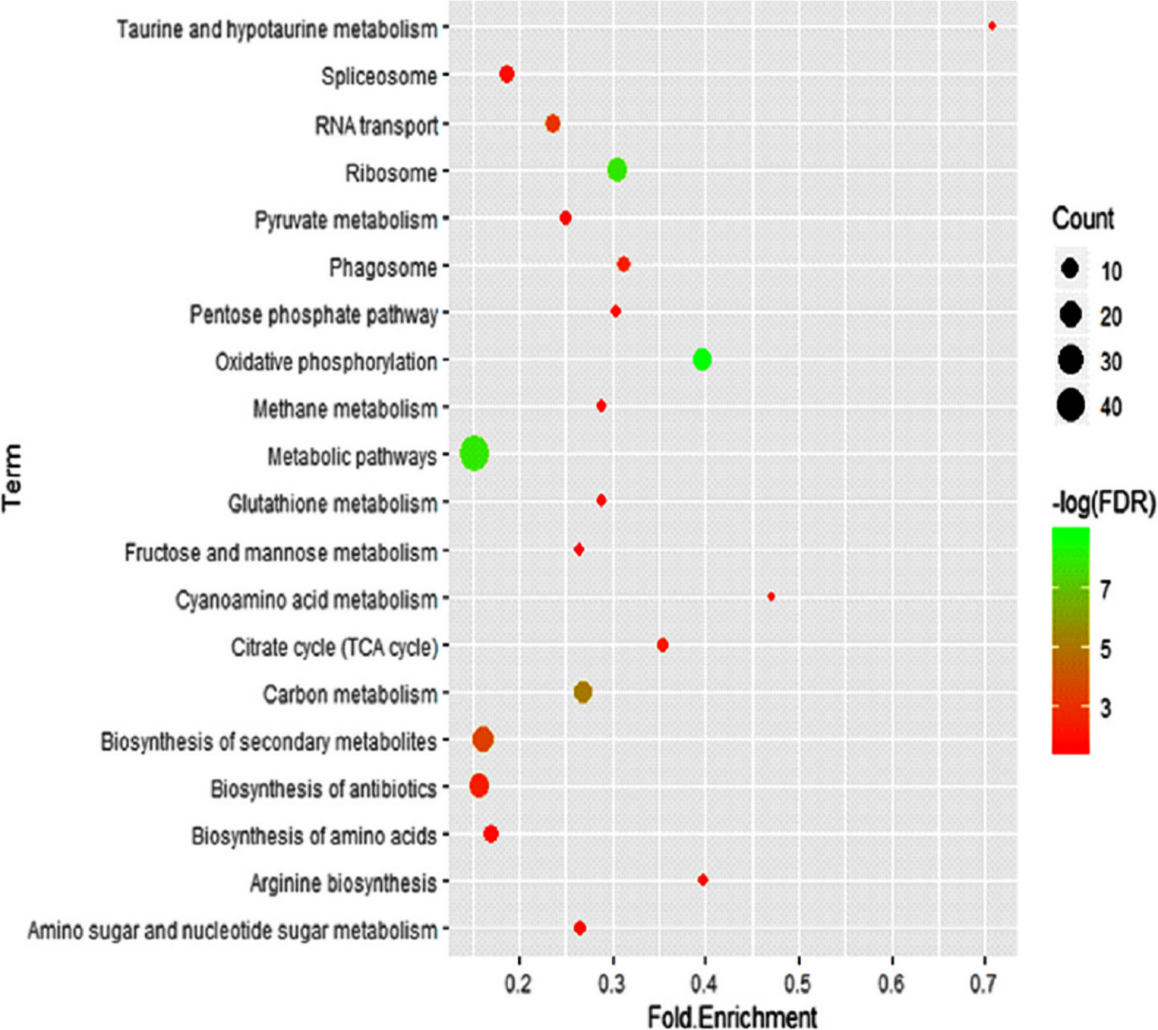
Enrichment Analysis of differential proteins by KEGG metabolic pathway. Notes: The circle was used to represent the enrichment of each KEGG term, the diameter of the circle represented the number of differentially expressed genes commented to each KEGG term species, the color of the circle represented the significance of the corresponding KEGG term (p value corrected by FDR). Bottom transverse coordinate represented “Rich factor”, the larger the value, the higher the proportion of the number of differentially expressed genes to the total number of corresponding KEGG term gene

The information of GO term and KEGG metabolic pathway analysis indicated that the enrichment of differential expressed proteins between VGI and VGII was mainly directed to Golgi apparatus, Golgi membrane and Golgi vesicle as shown in Table 3. The most significant enriched metabolic pathways were oxidative phosphorylation, ribosome and metabolic pathway as shown in Fig.6.

**Table 3:** Results of GO enrichment analysis for Cellular Component

GO ID	GO Term	Gene Ratio	Bg Ratio	*P* value
GO:0005794	Golgi apparatus	40	51	3.26E-07
GO:0000139	Golgi membrane	26	32	1.37E-05
GO:0005798	Golgi-associated vesicle	12	16	0.011314374
GO:0031982	vesicle	17	26	0.021147847
GO:0030662	coated vesicle membrane	14	21	0.028770779
GO:0044433	cytoplasmic vesicle part	15	23	0.030922717
GO:0034708	methyltransferase complex	4	4	0.036668632
GO:0030120	vesicle coat	12	18	0.042486706
GO:0098796	membrane protein complex	50	95	0.046997979
GO:0030135	coated vesicle	14	22	0.047857677
GO:0031988	membrane-bounded vesicle	15	24	0.049777137

## Discussion

### Selection of identification method and drug resistance of Chinese *C. gattii* strains

*C. neoformans* and *C. gattii* are two important fungal pathogens for humans and animals. Both of them are round with capsule and positive urease test. These routine laboratory identification methods are unable to distinguish the two species [7]. Although there were several reports about infection of *C. gattii* from China [8–10], the number of *C. gattii* infections may have been underestimated because most laboratories utilize ink staining and biochemical methods, like VITEK 2 compact, for routine identificatio. CGB agar was recommended to identify *C. gattii* from *C. neoformans* due to its convenience and low cost [7], but it took longer time to get the result. Molecular methods would be faster and more accurate for differentiation of the two pathogenic Cryptococci species. However, they are not available in all clinical laboratories, where an accurate identificaction of species should be made in order to install the proper treatment. In this study, eight strains of *C. gattii* were not only correctly identified but also grouped by MALDI-TOF MS with Bruker software 3.0. Of course, eight strains was not enough and more strains should be studied to obtain the spectrum difference between groups of *C. gattii*.

The results of antifungal susceptibility test by two kits were in good agreement, except that MICs of Fluconazole were 2~4 times higher by Yeast one than by ATB fungus 3. All the *C.gattii* isolates were susceptible to 5-Flucytosine, Amphotericin B, Itraconazole, Voriconazole and Posaconazole, only one strain was resistant to Fluconazole with MIC of 128 by Yeast one. However, interpretation of MICs of antifungal agents including Fluconazole for category of “susceptibility” or “resistance” was hampered by the lack of clinical breakpoints for *C. gattii*. Some previous studies have shown that *C. gattii* may be less susceptible to antifungal agents compared with *C. neoformans*. In a study from Taiwan, *C. gattii* was less susceptible to 5-Flucytosine and amphotericin B, and in Spain, MICs of Fluconazole, Voriconazole and Posaconazole to *C. gattii* were significantly higher [11–13]. The correlation between susceptibility profile and genotype of *C. gattii* has rarely been studied [14], our data indicated that antifungal agents exhibited higher MICs against isolates of genotype VGII than genotype VGI, which agreed with the data of Hagen et al. and Lockhart et al. [14–17]. However, Clinical relevance between MIC breakpoint and epidemiological cut-off value (ECV) based on MIC distributions of wild-type strains has currently been studied on both *C. neoformans* and *C. gattii* isolates from Europe, USA, Australia, Brazil, Canada, India and South Africa. ECVs of Amphotericin B (0.5~1 μg/ml), 5-Flucytosine (4~16 μg/ml), Fluconazole (8~32 μg/ml) and other azoles varied similarly by molecular type for both *C. neoformans* and *C. gatti*i [15, 16].

### Molecular and epidemical characteristics of Chinese *C. gattii* strains

Up to now, four genotypes of *C. gattii* have been detected, they were VGI, VGII, VGIII and VGIV. Lockhart and colleagues had reported that VGII and VGIII were the most-frequently identified isolates in America, VGIV was almost exclusively in Africa, and VGI predominated in Europe, Australia and Asia [18]. *C. gattii*, as an important pathogen, caused outbreak in British Columbia, Canada and the Pacific Northwest, the United States. But 8 strains in our study were pathogens causing sporadic infections according to the strain origination and their isolation time.

### Patient information and clinical characteristics

All the patients in our study had no recent travel history, the *C. gattii* infection occurred regionally and domestically. They were immunocompetent young male adults with age range of 21~60 years old, which was considered as the reason for increased exposure to environmental sources and increased chance for infection [19]^.^. Most patients had headache and fever, three patients showed neck stiffness, positive Kernig’s sign and meningeal lesions, meningeal enhancement or lacunar infarction by brain MRI. Most of them also demonstrated irregular nodule, consolidation and mass by lung CT, which was consistent with the research made by Ngamskulrungroj who indicated that *C. gattii* could cause fatal lung infection [20].

It was reported that host response varied based on the genotype of the organism and concomitant illnesses [21]. There was also study which revealed that *C. gattii* VGII strains were more virulent than VGI strains and VGIIa were even more virulent than VGIIb independent of their clinical or environmental origin [22]. In our study, all patients were treated by Fluconazole plus amphotericin B, most of them improved effectively without severe neurological sequelae. This might be explained by the fact that these patients infected by *C. gattii* VGI more than *C. gattii* VGII on the one hand, and on the other hand, two of them were infected with *C. gattii* VGII which showed sequence mutations in the gene location of RAS1 and FTR1 compared with reference strains of R265 and R272 respectively. Whether the mutations were relevant to virulence decrease, further work needs to be done.

### Analysis of differentially expressed protein in two genotypes of *C. gattii*

Our study showed significant difference in protein expression spectra between VGI and VGII. GO is a very important tool of biological information. By establishing a set of controlled words with dynamic form, the attributes of genes and gene products in organisms can be described comprehensively. KEGG is the main public database with which the most important biochemical metabolic pathways and signal transduction pathways can be determined. Our results suggested that the differential protein of *C. gattii* VGI and VG II was mainly located on the organelles associated with the Golgi body, which meant energy metabolism *of C. gattii* might be involved in the difference of pathogenesis mechanism for different genotype of *C. gattii*. Study on the secretory protein and the secretory vesicles of the two genotypes of *C. gattii* would be beneficial to the understanding of the pathogenesis of *C. gattii*.

## Conclusions

In spite that only 8 strains were available for analysis, our results demonstrated that *C. gattii* genotype VGI was predominant in China and *C. gattii* genotype VGII was more resistant to antifungal agents. *C. gattii* VGII isolates possessed obvious protein peaks with molecular weight of approximate 4342, 8686, 9611 Da. The full protein spectrum data of *C.gattii* were provided for the first time, a total of 3628 proteins were identified, 329 and 180 proteins were highly expressed by *C. gattii* VGI and VGII respectively. The enrichment of differentially expressed proteins between VGI and VGII was mainly directed to Golgi complex. It will lay a foundation for better understanding and further research on the pathogenic mechanism for different genotype of *C. gattii*.

## Methods

### Collection and initial identification of clinical isolates

A total of 254 clinical isolates initially identified as *C. neoformans* by biochemical methods were collected from seven hospitals in China, they were all stored at − 80 °C. All the isolates were sub-cultured onto Sabouraud dextrose agar medium at 25 °C for 48~72 h and identified by VITEK 2 Compact (bioMérieux SA, France). Each isolate was also inoculated on canavanine-glycine-bromthymol blue (CGB) agar [23] at 25 °C for 24~120 h, The isolates which showed blue pigmentation on CGB agar were then subjected to further identification.

### Identification by MALDI-TOF MS

Isolates were re-identified and analyzed by MALDI-TOF MS (Bruker, Daltonik, Bremen, Germany). Briefly, one single colony was smeared directly on the MALDI-TOF MS analysis plate and formic acid was added. After 3~5 min, matrix was added and the plate was put into the MALDI-TOF MS instrument with MALDI Biotyper software 3.0 (Bruker Daltonik GmbH) for species identification and dendrogram analysis. Two strains of *C.neoformans* were included as outgroup control.

### Analysis by multilocus sequence typing (MLST)

Genomic DNA of each isolate identified as *C. gattii* was then extracted using the TianGen® TIANamp Yeast DNA Kit (Tiangen Biotech Beijing CO., LTD, China) complying with the manufacturer’s instruction, seven genes of unlinked loci were amplified, including six housekeeping genes (CAP59, GPD1, LAC1, PLB1, SOD1, URA5) and one non-coding region gene (IGS1) [24], bi-directional sequencing for each gene was then carried out, sequence comparison for each locus was done according to the method described by ISHAM Cryptococcal Working Group. Sequences were uploaded to the MLST Database for the *C.neoformans/C.gattii* species complex (http://mlst.mycologylab.org) one by one. A sequence type (ST) number and seven allele type (AT) numbers were given to each isolate. New AT and ST number will be assigned for new sequence. To differentiate VGII subtypes, twelve unlinked genes (SXI1α, TEF1, FTR1, CBP1, ICL1, HOG1, TOR1, STE7, TRR1, FHB1, RAS1, PAK1) were also amplified and sequenced according to the method of Fraser et al. [25], they were analyzed and compared with Vancouver Island strains of R265 and R272.

### Antifungal susceptibility test of *C. gattii* isolates

Antifungal susceptibility test was carried out by both ATB fungus 3 (bioMérieux SA, France) and Yeast one (Trek Diagnostic Systems Ltd., UK) following their instructions. *Candida krusei* ATCC 6258 and *Candida parapsilosis* ATCC 22019 were used for quality control.

### Clinical information of patients

Under authorization of Medical Record Department, Clinical information of patients infected by *C. gattii* was reviewed retrospectively, including age, sex, underlying diseases, symptoms, imaging findings, laboratory examinations and antifungal therapy.

### Analysis of differential proteins

According to the regional distribution and the integrity of clinical data of the strains, six strains (4 of VGI and 2 of VGII) of *C. gattii* were selected for proteome analysis. All the isolates were sub-cultured onto Sabouraud dextrose agar medium, incubated at 25 °C for 48~72 h , the proteins were extracted from the strains, The protein concentration was 0.5 μg/μl, after being separated by SDS-PAGE electrophoresis (One-dimensional), the proteins were hydrolyzed and analyzed. Original mass spectrum files of 6 strains were imported into Maxquatt software (version 1.3.0.5) for analysis. The database source was for *C.gattii*. in Uniprot (http://www.geneontology.org/). Plus or minus 15 ppm of polypeptide molecular weight, or missing 2 cutting sites were set as Maxquatt parameters, false discovery rate (FDR) for polypeptide identification was set as 0.01. A standard of *p* < 0.05 and 2-fold expression were considered as different proteins. The enrichment and pathway analysis was carried out by using the GO plot package of R language and KEGG online tool (https://david.ncifcrf.gov/, http://www.kegg.jp/kegg/pathway.html). The bubble diagram was drawn according to the R language ggplot2 package.

## Supplementary information

**Additional file 1: Supplementary Table 1**: MLST profiles and genotype of 8 *C. gattii* strains. **Supplementary Table 2**: MLST profiles of 16 gene loci for 2 *C. gattii* strains with genotype VGII. Notes: R265 and R272 were Vancouver Island reference strains for *C. gattii* genotype VGIIa and VGIIb respectively; / represented that data was not available; GenBank accession numbers for multilocus sequence typing alleles were: CAP59–1, DQ096432; CAP59–2, DQ096433; GPD1–1, DQ096377; GPD1–6, DQ096382; IGS-4, DQ096314; IGS-10, DQ096319; PLB1–1, DQ096343; PLB1–2, DQ096344; SXI1α-18, DQ096308; SXI1α-19, AY973646; TEF1–7, DQ096364; TEF1–5, DQ096362; FTR1–1, DQ096448; FTR1–2, DQ096449; CBP1–1, DQ096435; CBP1–2, DQ096436; ICL1–1, DQ096458; ICL1–2, DQ096459; HOG1–1, DQ096456; TOR1–1, DQ096470; STE7–1, DQ096467; STE7–2, DQ096468;TRR1–1, DQ096472; TRR1–2, DQ096473; RAS1–1, DQ096464; RAS1–2, DQ096465; PAK1–1, DQ096461; PAK1–2, DQ096462.

## Data Availability

The datasets used and/or analyzed during the current study are available from the corresponding author and first author on reasonable request.

## Abbreviations

AMB: Amphotericin B
AT: Allele type
CGB: Canavanine glycine bromothymol blue
*C. gattii*: *Cryptococcus gattii*
*C. neoformans*: *Cryptococcus neoformans*
*ECVs*: Epidemiological MIC cut-off values
FCA: Fluconazole
FDR: False discovery rate
GO: Gene ontology
ITR: Itraconazole
KEGG: Kyoto encyclopedia of genes and genomes
MALDI-TOF MS: Matrix-assisted laser desorption/ionization time-of-flight mass spectrometry
MICs: Minimal inhibitory concentrations
MLST: Multi-locus sequence typing
PSZ: Posaconazole
ST: Sequence type
VRC: Voriconazole
5-FU: 5-Flucytosine
