# Supplementary material for: Clinical and microbiological characteristics of Cryptococcus gattii isolated from 7 hospitals in China
Source: BMC Microbiol. 2020 Mar 30;20:73. doi: 10.1186/s12866-020-01752-4 (PMC7106762; doi:10.1186/s12866-020-01752-4)
Supplement: Supplementary file 1 — Additional file 1: Supplementary Table 1: MLST profiles and genotype of 8 C. gattii strains. Supplementary Table 2: MLST profiles of 16 gene loci for 2 C. gattii strains with genotype VGII. Notes: R265 and R272 were Vancouver Island reference strains for C. gattii genotype VGIIa and VGIIb respectively; / represented that data was not available; GenBank accession numbers for multilocus sequence typing alleles were: CAP59–1, DQ096432; CAP59–2, DQ096433; GPD1–1, DQ096377; GPD1–6, DQ096382; IGS-4, DQ096314; IGS-10, DQ096319; PLB1–1, DQ096343; PLB1–2, DQ096344; SXI1α-18, DQ096308; SXI1α-19, AY973646; TEF1–7, DQ096364; TEF1–5, DQ096362; FTR1–1, DQ096448; FTR1–2, DQ096449; CBP1–1, DQ096435; CBP1–2, DQ096436; ICL1–1, DQ096458; ICL1–2, DQ096459; HOG1–1, DQ096456; TOR1–1, DQ096470; STE7–1, DQ096467; STE7–2, DQ096468;TRR1–1, DQ096472; TRR1–2, DQ096473; RAS1–1, DQ096464; RAS1–2, DQ096465; PAK1–1, DQ096461; PAK1–2, DQ096462. [file 12866_2020_1752_MOESM1_ESM.docx]

**Supplementary Table 1 MLST profiles and Genotype of 8 *C. gattii* strains**

| isolate No. | Multilocus sequence typing profile (Allele number) | | | | | | | STs | Genotype |
| --- | --- | --- | --- | --- | --- | --- | --- | --- | --- |
|  | CAP59 | GPD1 | IGS1 | LAC1 | PLB1 | SOD1 | URA5 |  |  |
| 1 | 1 | 1 | 4 | 4 | 1 | 14 | 7 | 20 | VGII |
| 2 | 36 | 11 | 13 | 5 | 13 | 36 | 14 | 106 | VGI |
| 3 | 16 | 5 | 3 | 5 | 5 | 65 | 12 | 57 | VGI |
| 4 | 16 | 11 | 46 | 13 | 13 | 34 | 15 | 197 | VGI |
| 5 | 16 | 5 | 3 | 5 | 5 | 65 | 12 | 57 | VGI |
| 6 | 2 | 6 | 10 | 4 | 2 | 15 | 2 | 7 | VGII |
| 7 | 16 | 5 | 3 | 5 | 5 | 65 | 12 | 57 | VGI |
| 8 | 16 | 33 | 44 | 13 | 13 | 47 | 24 | 161 | VGI |

**Supplementary Table 2 MLST profiles of 16 gene loci for 2 *C. gattii* strains with genotype VGII**

| isolate NO. | Multilocus sequence typing profile (Allele number) | | | | | | | | | | | | | | | |
| --- | --- | --- | --- | --- | --- | --- | --- | --- | --- | --- | --- | --- | --- | --- | --- | --- |
|  | **CAP59** | **GPD1** | **IGS1** | **PLB1** | **SXI1α** | **TEF1** | **FTR1** | **CBP1** | **ICL1** | **HOG1** | **TOR1** | **STE7** | **TRR1** | **FHB1** | **RAS1** | **PAK1** |
| R265 | 1 | 1 | 4 | 1 | 18 | 7 | 1 | 1 | 1 | 1 | 1 | 1 | 1 | 1 | 1 | 1 |
| 1 | 1 | 1 | 4 | 1 | / | 7 | / | 1 | 1 | 1 | 1 | 2 | 1 | 1 | / | 1 |
| R272 | 2 | 6 | 10 | 2 | 19 | 5 | 2 | 2 | 2 | 1 | 1 | 2 | 2 | 1 | 2 | 2 |
| 6 | 2 | 6 | 10 | 2 | / | / | / | 2 | 2 | 1 | 1 | 2 | 2 | 1 | / | / |

Notes: R265 and R272 were Vancouver Island reference strains for *C. gattii* genotype VGIIa and VGIIb respectively; / represented that data was not available; GenBank accession numbers for multilocus sequence typing alleles were: CAP59-1, DQ096432; CAP59-2, DQ096433; GPD1-1, DQ096377; GPD1-6, DQ096382; IGS-4, DQ096314; IGS-10, DQ096319; PLB1-1, DQ096343; PLB1-2, DQ096344; SXI1α-18, DQ096308; SXI1α-19, AY973646; TEF1-7, DQ096364; TEF1-5, DQ096362; FTR1-1, DQ096448; FTR1-2, DQ096449; CBP1-1, DQ096435; CBP1-2, DQ096436; ICL1-1, DQ096458; ICL1-2, DQ096459; HOG1-1, DQ096456; TOR1-1, DQ096470; STE7-1, DQ096467; STE7-2, DQ096468;TRR1-1, DQ096472; TRR1-2, DQ096473; RAS1-1, DQ096464; RAS1-2, DQ096465; PAK1-1, DQ096461; PAK1-2, DQ096462.
